# Rapid kinetics reveal surprising flavin chemistry in bifurcating electron transfer flavoprotein from *Acidaminococcus fermentans*

**DOI:** 10.1074/jbc.RA120.016017

**Published:** 2020-12-02

**Authors:** Jeerus Sucharitakul, Wolfgang Buckel, Pimchai Chaiyen

**Affiliations:** 1 Department of Biochemistry, Chulalongkorn University, Patumwan, Bangkok, Thailand; 2 Skeletal Disorders Research Unit, Faculty of Dentistry, Chulalongkorn University, Patumwan, Bangkok, Thailand; 3 Laboratorium für Mikrobiologie, Fachbereich Biologie and Synmikro, Philipps-Universität, Marburg, Germany; 4 Max-Plank-Institut für terrestrische Mikrobiologie, Marburg, Germany; 5 School of Biomolecular Science and Engineering, Vidyasirimedhi Institute of Science and Technology (VISTEC), Rayong, Thailand

**Keywords:** flavin-based electron bifurcation, *Acidaminococcus fermentans*, electron transfer flavoprotein, anionic FAD semiquinone, rapid kinetics, inverse isotope effect, ASQ, anionic semiquinone of FAD, FAD^•−^, Bcd, butyryl-CoA dehydrogenase, EtfAB, Etf (electron transfer flavoprotein) containing α-FAD bound on A- and β-FAD bound on B-subunit, EtfaB, Etf containing only β-FAD, Fd, ferredoxin, Fld, flavodoxin, HQ, hydroquinone form of FAD, FADH⁻, Q, quinone form of FAD

## Abstract

Electron bifurcation uses free energy from exergonic redox reactions to power endergonic reactions. β-FAD of the electron transfer flavoprotein (EtfAB) from the anaerobic bacterium *Acidaminococcus fermentans* bifurcates the electrons of NADH, sending one to the low-potential ferredoxin and the other to the high-potential α-FAD semiquinone (α-FAD^•−^). The resultant α-FAD hydroquinone (α-FADH^−^) transfers one electron further to butyryl-CoA dehydrogenase (Bcd); two such transfers enable Bcd to reduce crotonyl-CoA to butyryl-CoA. To get insight into the mechanism of these intricate reactions, we constructed an artificial reaction only with EtfAB containing α-FAD or α-FAD^•−^ to monitor formation of α-FAD^•−^ or α-FADH^−^, respectively, using stopped flow kinetic measurements. In the presence of α-FAD, we observed that NADH transferred a hydride to β-FAD at a rate of 920 s^−1^, yielding the charge–transfer complex NAD^+^:β-FADH^−^ with an absorbance maximum at 650 nm. β-FADH^−^ bifurcated one electron to α-FAD and the other electron to α-FAD of a second EtfAB molecule, forming two stable α-FAD^•−^. With α-FAD^•−^, the reduction of β-FAD with NADH was 1500 times slower. Reduction of β-FAD in the presence of α-FAD displayed a normal kinetic isotope effect (KIE) of 2.1, whereas the KIE was inverted in the presence of α-FAD^•−^. These data indicate that a nearby radical (14 Å apart) slows the rate of a hydride transfer and inverts the KIE. This unanticipated flavin chemistry is not restricted to Etf–Bcd but certainly occurs in other bifurcating Etfs found in anaerobic bacteria and archaea.

Electron bifurcation sends one electron of a donor pair to an acceptor with a higher reduction potential (low energy level) and the other to an acceptor with a lower reduction potential (high energy level) (1, 2, 3, 4, 5). Nature uses the high energy electron to establish ion gradients for energy conservation or to conduct difficult reductions such as nitrogen fixation or synthesis of molecular hydrogen. In the so-called flavin-based electron bifurcation, the reductant NAD(P)H reduces FAD to the hydroquinone state (HQ, FADH^−^), which bifurcates one electron to an acceptor with a higher reduction potential, such as crotonyl-CoA, heterodisulfide, menaquinone, or benzoquinone, and the other to ferredoxin (Fd) or flavodoxin (Fld), which have reduction potentials between −390 and −420 mV (6).

Recently, we measured the reduction potentials of a bifurcating system composed of electron transfer flavoprotein (electron transfer flavoprotein containing α-FAD bound on A- and β-FAD bound on B-subunit [EtfAB]) and butyryl-CoA dehydrogenase (Bcd); both proteins are from the anaerobic gut bacterium *Acidaminococcus fermentans* (7, 8, 9, 10, 11). In contrast to Etfs involved in the β-oxidation of fatty acids, which only contains α-FAD that can stabilize an anionic semiquinone (ASQ), the *A. fermentans* EtfAB harbors an additional bifurcating β-FAD which is bound to the B-subunit. We propose that NADH ($E^{{}^{\circ}}\prime$ = −320 mV) reduces β-FAD ($E^{{}^{\circ}}\prime$ = −271 mV) by transferring a hydride to generate β-FADH^˗^, which bifurcates one electron energetically uphill from its ASQ/HQ potential of +172 mV to α-FAD^•−^($E^{{}^{\circ}}\prime$ = −36 mV) and the other electron downhill from its Q/ASQ potential of −714 mV to Fd ($E^{{}^{\circ}}\prime$ = −390 mV). The formed α-FADH^−^ on the rotatable domain II of Etf makes a turn by 90° and encounters δ-FAD of Bcd (10), whereby its reduction potentials change to a two-electron potential of $E^{{}^{\circ}}\prime$ = −228 mV, almost the same as that of β-FAD, which allows a smooth further one-electron transfer to δ-FAD to generate δ-FAD^•−^ ($E^{{}^{\circ}}\prime$ = +162 mV). The electron derived from the next bifurcation round reduces δ-FAD^•−^ further to δ-FADH⁻ ($E^{{}^{\circ}}\prime$ = −63 mV). The formed δ-FADH⁻ changes its reduction potentials to the two-electron potential of −100 mV and reduces crotonyl-CoA by hydride transfer to butyryl-CoA ($E^{{}^{\circ}}\prime$ = −10 mV) (11).

To get insight into the mechanism of the electron bifurcation, stopped-flow kinetics were applied. However, the complete reaction turned out to be much too fast for this method. The red anionic semiquinone of α-FAD occurred already before the dead time of the instrument (1 ms). Therefore, Fd and Bcd were omitted, and only the slower reduction of EtfAB with NADH was investigated. To get adjusted to the system, the experiments started with Etf containing only β-FAD (EtfaB), from which α-FAD was removed. The main part of this work consists of the reduction mechanism of EtfAB with NADH and (4*R*)[4-^2^H]NADH (NADD). Though still very fast, the rate constant of the first reduction of β-FAD with NADH could be determined, whereas the second reduction occurred 1500 times slower. The slow reduction was caused by the neighboring α-FAD when it was in the α-FAD^•−^-redox state. Interestingly, reduction of EtfAB by NADD exhibited a normal kinetic isotope effect (KIE), whereas in the presence of α-FAD^•−^, the KIE became inverse.

## Results

### Reduction kinetics of EtfaB

#### Reduction of EtfaB by a stoichiometric amount of NADH

**Figure 1:**
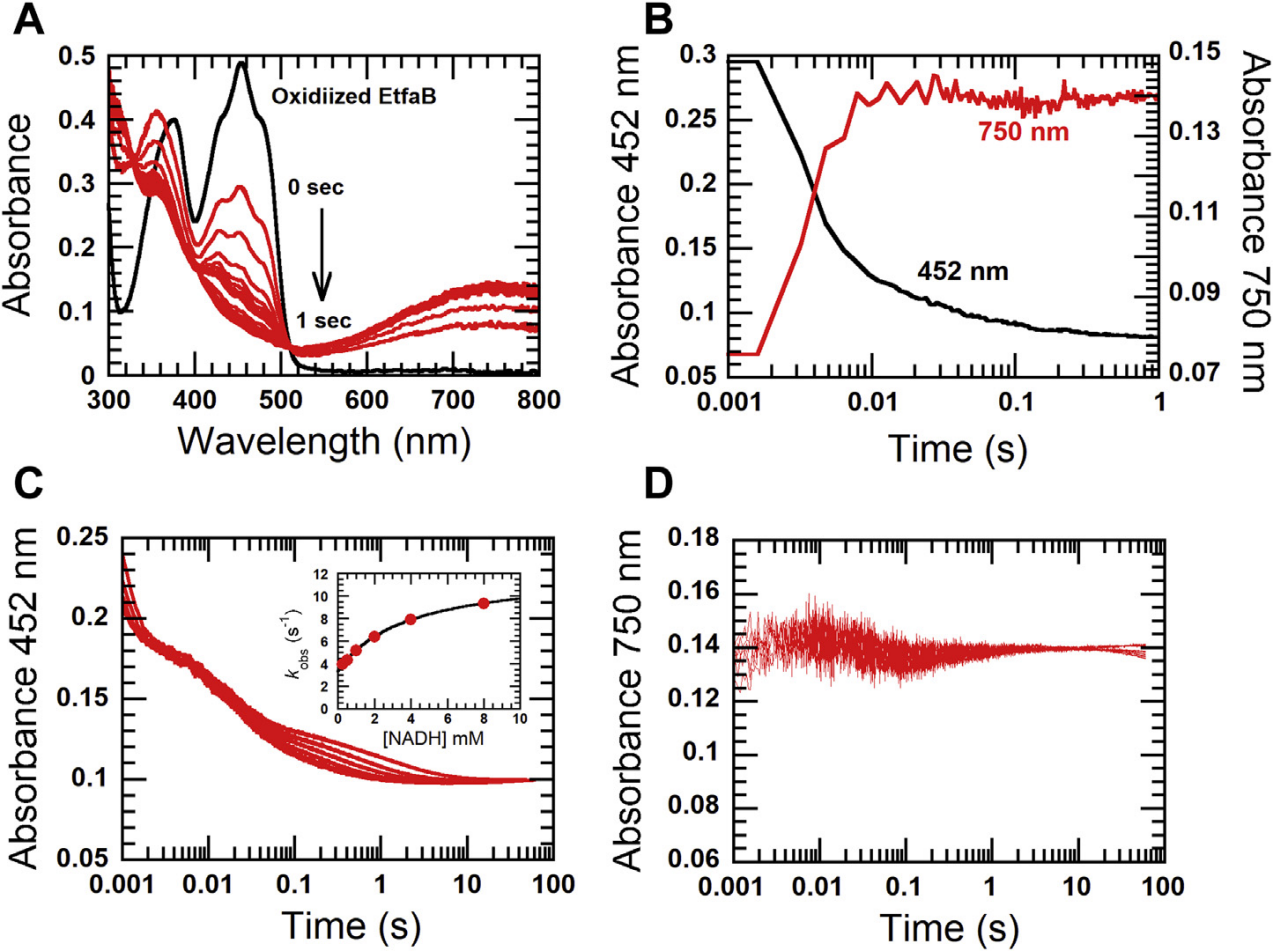
**Reduction of EtfaB with NADH.***A*, EtfaB (45 μM, *black line*) was mixed with 45 μM NADH and observed for 1 s (*red lines*). *B*, the wavelengths at 452 nm (FAD, *black line*) and 750 nm (charge–transfer complex, *red line*) were selected from the CCD spectra in *A*. *C*, 50 μM EtfAB was mixed with 0.25, 0.5, 1, 2, 4 and 8 mM NADH. The reactions were monitored for flavin reduction at 452 nm. The uppermost kinetic trace to the lowest trace were from the lowest to the highest NADH concentrations. The analysis of the kinetic traces at 452 nm shows two exponential phases for fast reduction (before 0.001 s to 0.01 s) and slow reduction (0.01–1 s). The inset shows a plot of the observed rate constants of the slow reduction *versu*s NADH concentrations. *D*, all kinetic traces of the absorbance at 750 nm from varied NADH concentrations are superimposed. CCD, charge coupled device array; EtfAB, Etf (electron transfer flavoprotein) containing α-FAD bound on A- and β-FAD bound on B-subunit; EtfaB, Etf containing only β-FAD.

The stoichiometric reduction of 45 μM EtfaB (containing 45 μM β-FADH but no α-FAD (11) with 45 μM NADH resulted in two-phase kinetics. About 40% of the reduction of EtfaB (from *black line to the first red line*, Fig. 1*A*) was fast and occurred before the dead time (∼0.002 s) of the stopped-flow machine (ΔA_452_ ∼0.20 at 0.002 s, Fig. 1*B*). The remaining oxidized EtfaB (60%) was reduced within 0.002 and 1.0 s (*red lines*, Fig. 1*A* and *black line*, Fig. 1*B*). The absorbance at 340 nm (*red lines*, Fig. 1*A*) was from residual NADH, which completely disappeared after 1 s. During 0.002 and 0.01 s, the absorbance at 750 nm increased (*red line*, Fig. 1*B*), probably because of formation of the β-FADH^−^:NAD^+^ charge–transfer complex found in many flavoproteins (12). During the whole reduction process, the characteristic spectrum of a red semiquinone did not show up (absorbance increase around 360–370 nm). The kinetic trace monitored at A_452_ could be fitted with two exponential phases. The first phase (dead time −0.01 s) occurred concurrently with the charge–transfer complex formation which showed a rate constant of 213 s^−1^, whereas the second phase (0.01–1.0 s) showed about 19-times slower rate constant of 11 ± 0.2 s^−1^.

#### Reduction of EtfaB by NADH under pseudo-first order conditions

Reduction of 50 μM EtfaB by various concentrations of NADH ranging from 0.25 mM to 8 mM (Fig. 1*C*) was carried out and monitored by stopped-flow spectrophotometry. The reduction kinetics showed two exponential phases with a fast exponential phase (dead time −0.006 s) and a slow exponential phase (0.006–20 s). The analysis of the observed rate constants for reduction (*k*_obs1_ and *k*_obs2_) which are dependent on NADH concentrations for reduction is according to Equation 1.

(1) $$A_{t}=A_{1}e^{k_{obs1}t}+A_{2}e^{-k_{obs2}t}+c$$

*A*_t_ is absorbance change at 452 nm at a given time. *A*_1_ and *A*_2_ are magnitude of absorbance changes of fast and slow reduction population, respectively. *C* is nonzero baseline.

**Figure 2:**
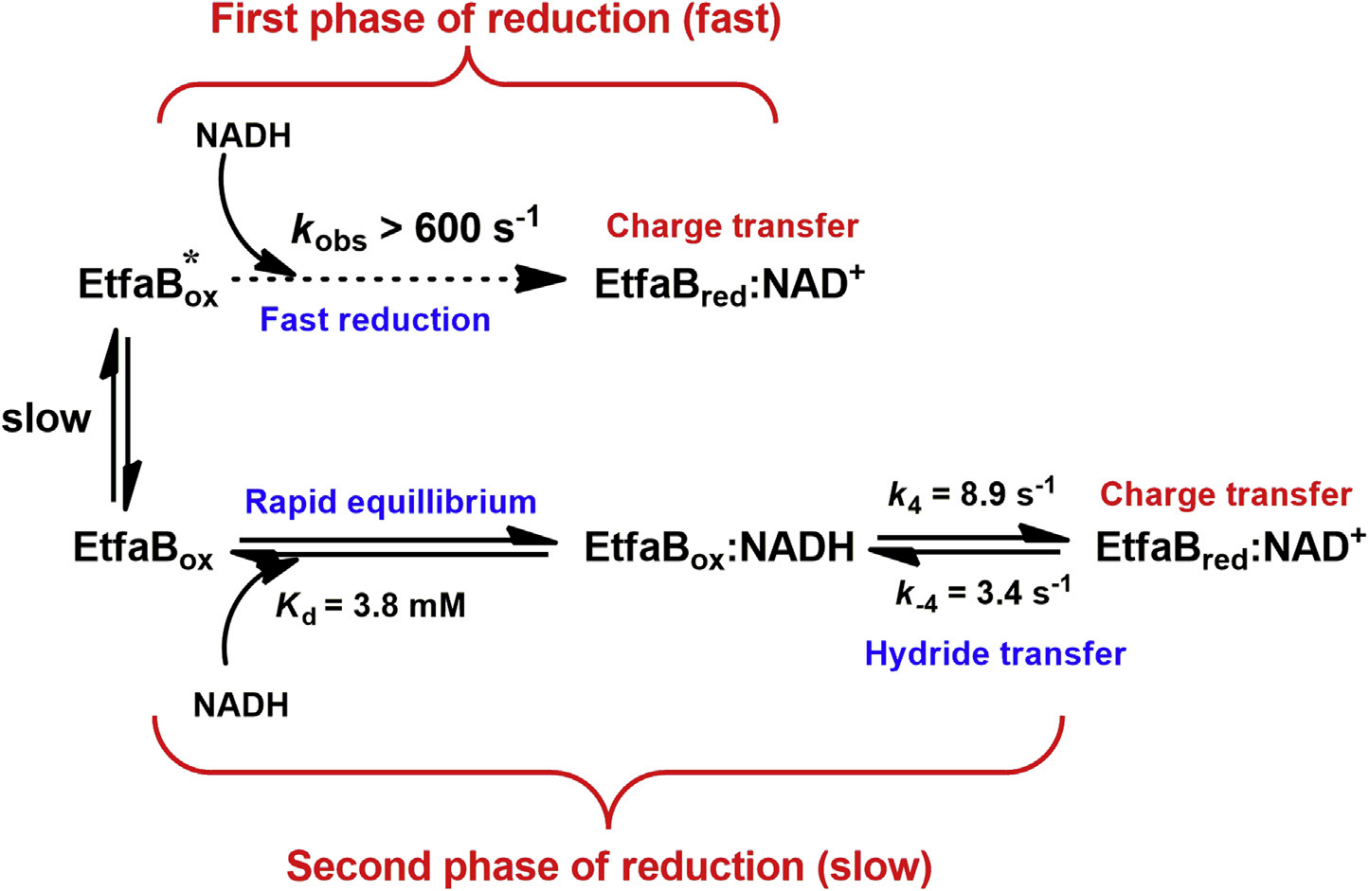
**The proposed kinetic mechanism for the reduction of EtfaB with NADH.** EtfaB contains two populations which exhibit fast (EtfaB∗) and slow (EtfaB) reduction. Both populations are proposed to be in slow equilibrium exchange. The mechanism of the slow reduction population is a two-step process, rapid binding of NADH on the oxidized enzyme to form the Michaelis–Menten complex, EtfaB_ox_:NADH, following a reversible hydride transfer to form the charge–transfer complex of reduced enzyme and NAD^+^ (EtfaB_red_:NAD^+^). EtfaB, Etf containing only β-FAD.

The fast species (*A*_1_, Equation 1) was the major enzyme population (∼84%, calculated from the starting A_452_ = 0.5 of the oxidized enzyme, Fig. 1*A*) which was reduced before 0.002 s, indicating that observed rate constants (*k*_obs_) for reduction was greater than 600 s^−1^ (EtfaB_ox_∗, Fig. 2). This phase also occurred concurrently with the charge–transfer complex formation at 750 nm (Fig. 1*D*). Another enzyme fraction (*A*_2_, Equation 1) was a minor enzyme population (∼16%) and less active (EtfaB_ox_, Fig. 2). Because the ratios of the absorbance changes at 452 nm of fast and slow kinetic phases were the same (∼4.6) from the lowest to the highest NADH concentration (Fig. 1*C*), the data suggest that both fast and slow reacting enzyme populations are linked by a very slow equilibrium and that both reductions proceeded independently from each other. The proposed mechanism is based on the two distinct kinetic properties of the fast and slow reduction populations also found in other flavoprotein systems (13, 14).

Because the kinetics of the fast reacting species mainly occurred during the dead time of the stopped-flow machine, we could only analyze the data of the slower phase. Kinetic analysis of the traces at 452 nm of the slow reacting species from the lowest to the highest NADH concentration showed two exponential phases (Fig. 1*C*). A plot of the observed rate constants as a function of the NADH concentration exhibited saturation kinetics according to Equation 2 with a significant intercept of 3.35 ± 0.05 s^−1^ (Inset in Fig. 1*C*). According to the model shown in Figure 2, the first step was treated as rapid binding of NADH to oxidized EtfaB_ox_ forming a Michaelis–Menten complex with *K*_d_ = 3.8 ± 0.2 mM. The second step was the reversible hydride transfer with a forward rate constant of 8.88 ± 0.15 s^−1^ (*k*_4_, Fig. 2) and a reverse rate constant of 3.35 ± 0.05 s^−1^ (*k*_−4_, Fig. 2).

(2) $$k_{obs}=\frac{k_{4}\left[NADH\right]}{K_{d}\;+\;\left[NADH\right]}\;+\;k_{-4}$$

### Reduction kinetics of the holoenzyme (EtfAB)

#### Reduction of EtfAB by an amount of NADH equivalent to β-FAD

**Figure 3:**
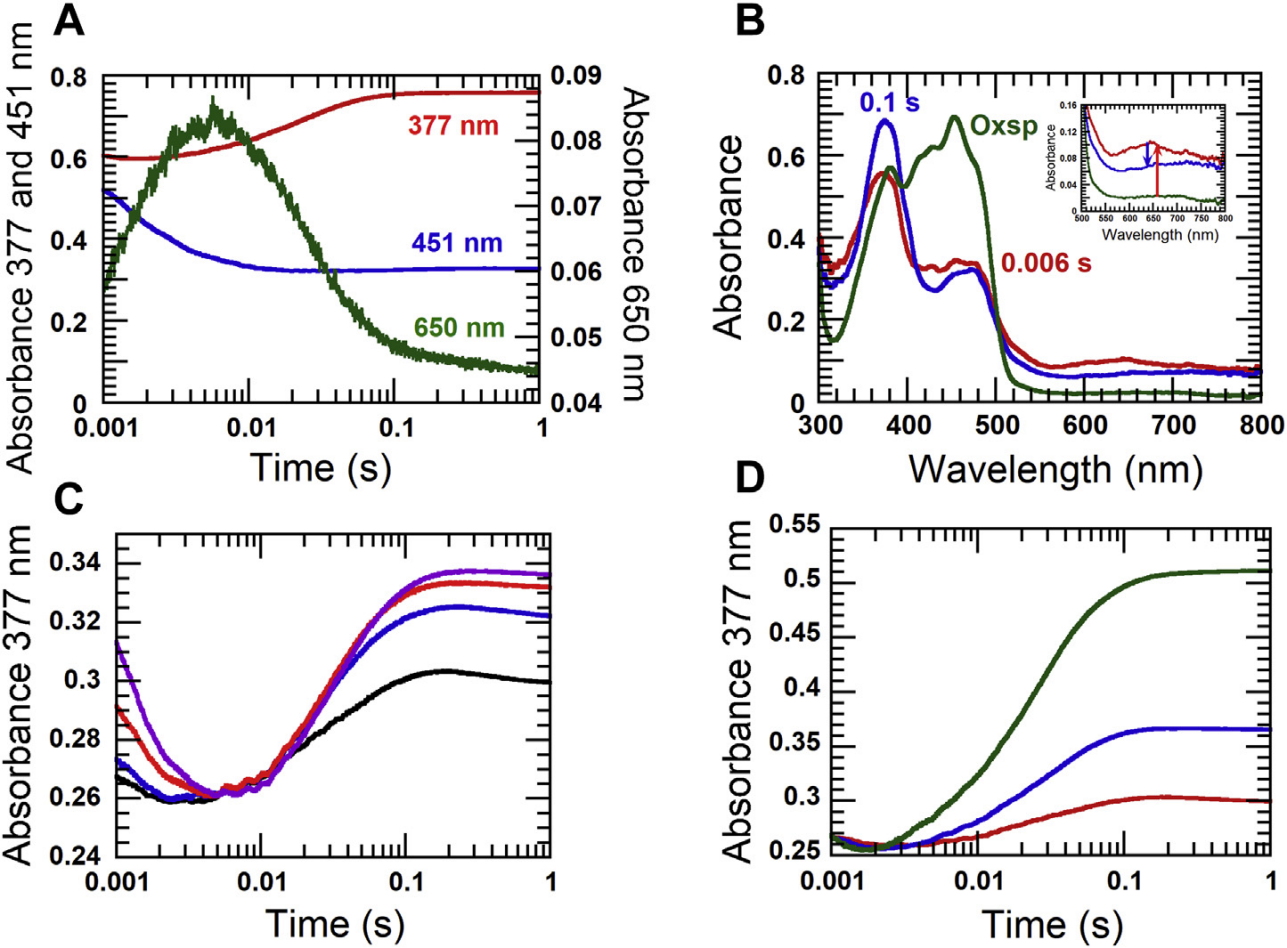
**Reduction of EtfAB with NADH equivalent to β-FAD.***A*, 34 μM EtfAB was mixed with 34 μM NADH. The reaction was monitored at 451 nm for flavin reduction (*blue line*), 377 nm for flavin red semiquinone (*red line*), and 650 nm for charge–transfer complex formation (*green line*). *B*, the whole spectrum of the same reaction (*A*) was taken before mixing with 34 μM NADH (*green line*); after mixing at 0.006 s (*red line*) and 0.1 s (*blue line*). Inset in *B* shows the magnified axes scale to focus the change of absorbance of charge transfer. The *red arrow* (*inset*) indicates the maximum of charge transfer. The *blue arrow* (*inset*) indicates a decay of charge transfer during electron bifurcation. *C*, the preequilibrated solution of 15 μM EtfAB was mixed with increasing EtfaB and NADH concentrations: 0 μM EtfaB +15 μM NADH (*black line*); 5 μM EtfaB +20 μM NADH (*blue line*); 15 μM EtfaB +30 μM NADH (*red line*), and 30 μM EtfaB +45 μM NADH (*purple line*). *D*, 15 μM EtfAB (*red line*), 30 μM EtfAB (*blue line*), and 60 μM EtfAB (*green line*) were mixed with anaerobic solutions of 15 μM, 30 μM, and 60 μM NADH, respectively. EtfAB, Etf (electron transfer flavoprotein) containing α-FAD bound on A- and β-FAD bound on B-subunit; EtfaB, Etf containing only β-FAD.

EtfAB (34 μM β-FAD) was reduced by 34 μM NADH and monitored at 451 nm using the stopped-flow spectrophotometer. The first phase (dead time −0.006 s) was a fast reduction with an observed rate constant of ∼450 s^−1^, whereby some part of the reaction occurred during the dead time (*blue line*, Fig. 3*A*). Concurrently, the charge–transfer complex β-FADH^−^:NAD^+^ (absorbance at 650 nm) was also formed (*green line*, Fig. 3*A*) during this phase. When compared with the data in Figure 1*A*, the observed rate constant of the stoichiometric reduction of the holo EtfAB was faster than that of EtfaB. This implies that the presence of α-FAD possibly affects the surroundings of β-FAD which accelerates the reduction rate constant. The second phase (0.006–0.1 s) was an increase in absorbance at 377 nm with the observed rate constant of 47 ± 1 s^−1^, because of red semiquinone formation. During this phase, the absorbance at 650 decreased, indicating the decay of the charge–transfer complex. It should be mentioned that the charge–transfer complex of EtfAB (absorbs maximally at 650 nm) was different from that of EtfaB which exhibits maximum absorbance at 750 nm. Because the initial absorbance (A_452_) of EtfAB used was ∼0.7 (*green line*, Fig. 3*B*), β-FAD was fully reduced by NADH (*red line*, Fig. 3*B*) at 0.006 s when ΔA_452_ was ∼0.35 (equivalent to the concentration of β-FAD bound to EtfAB). At 0.1 s, the stable semiquinone of α-FAD was formed (α-FAD^•−^) as represented by the blue line (Fig. 3*B*).

**Figure 4:**
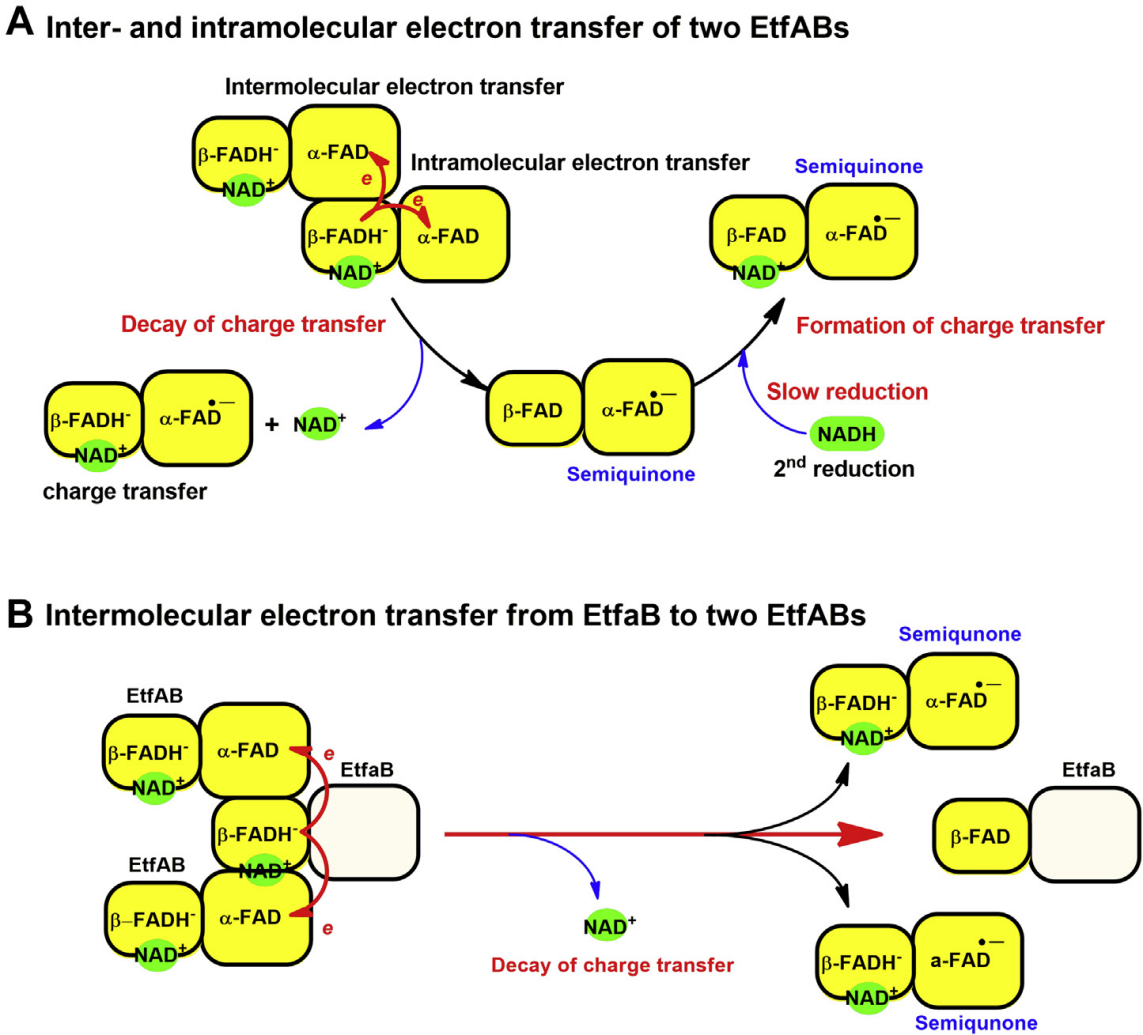
**Intramolecular and intermolecular electron transfer in EtfAB and EtfaB to EtfAB.***A*, after the first reduction, one β-FADH^−^ bifurcates one electron to α-FAD in the same Etf molecule and one electron to α-FAD of another Etf molecule. The electron bifurcation (as indicated by *coupled red arrows*) occurs simultaneously with the decay of the charge–transfer complex to form two different species of Etf with α-FAD^•−^. The second reduction of the re-oxidized β-FAD is strongly inhibited because α-FAD exists in semiquinone state. *B*, reduced EtfaB, an EtfAB without α-FAD, bifurcates two electrons to two α-FAD *via* intermolecular electron transfer to form two EtfAB with stable semiquinones (α-FAD^•−^) and one oxidized EtfaB. EtfAB, Etf (electron transfer flavoprotein) containing α-FAD bound on A- and β-FAD bound on B-subunit; EtfaB, Etf containing only β-FAD.

Under absence of a low-potential acceptor such as Fd or Fld, β-FADH^−^ most likely bifurcated one electron intramolecularly to α-FAD of the same EtfAB molecule and the other intermolecularly to α-FAD of another EtfAB molecule (Fig. 4*A*, as indicated by *red arrow*). This re-oxidation of β-FADH^−^ by intramolecular and intermolecular electron transfers was consistent with the decrease of the charge–transfer complex at 650 nm because of NAD^+^ release (from *red to blue line*, inset in Fig. 3*B*). During the first phase, the formation of 34 μM β-FADH^−^ caused an absorbance increase at 650 nm (*red line*, Fig. 3*B*) with a maximum ΔA_650_ ∼ 0.083 (*red arrow*, inset of Fig. 3*B*). Afterward, the absorbance decreased by ΔA_650_ ∼ 0.034 (*blue arrow*, inset of Fig. 3*B*). This amount of charge–transfer species was equivalent to 34 × 0.034/0.083 = 14 μM β-FADH^−^ which could generate 28 μM of stable α-FAD^•−^. These data indicate that only around 41% of β-FADH^−^ generated α-FAD^•−^ by electron bifurcation, whereas 59% of β-FADH^−^ remained reduced without transferring an electron.

To demonstrate that an electron from EtfAB-bound β-FADH^−^ can be transferred intermolecularly to another enzyme-bound α-FAD, EtfaB (+NADH) was added to increase the concentration of β-FADH^−^ without changing the concentration of α-FAD. An increase of β-FAD^−^ could mediate more intermolecular electron transfer. Hence, more red semiquinone should be detected according to more EtfaB concentrations. The data clearly indicate an increase in the amount of α-FAD^•−^ (noted by the increase in absorbance at 377 nm) without affecting its rate of formation (Fig. 3*C*). Interestingly, the generation of α-FAD^•−^ by electron bifurcation from β-FADH^−^ in EtfaB required the interaction of three molecules, EtfaB as donor with two EtfAB as electron acceptors (Fig. 4*B*, as indicated by *red arrows*). Owing to the lack of additional α-FAD, the further raise in EtfaB concentration resulted in saturation of the amount of α-FAD^•−^. In another experiment, in which the EtfAB and NADH concentrations were raised, the rate and amount of the generated α-FAD^•−^ raised proportionally, and no saturation was observed (Fig. 3*D*). The observed rate constants of 47 ± 4 s^−1^ for α-FAD^•−^ formation were similar at all EtfAB concentrations and similar to that of the red semiquinone formation in Figure 3*A*.

#### First and second reduction of β-FAD in EtfAB

**Figure 5:**
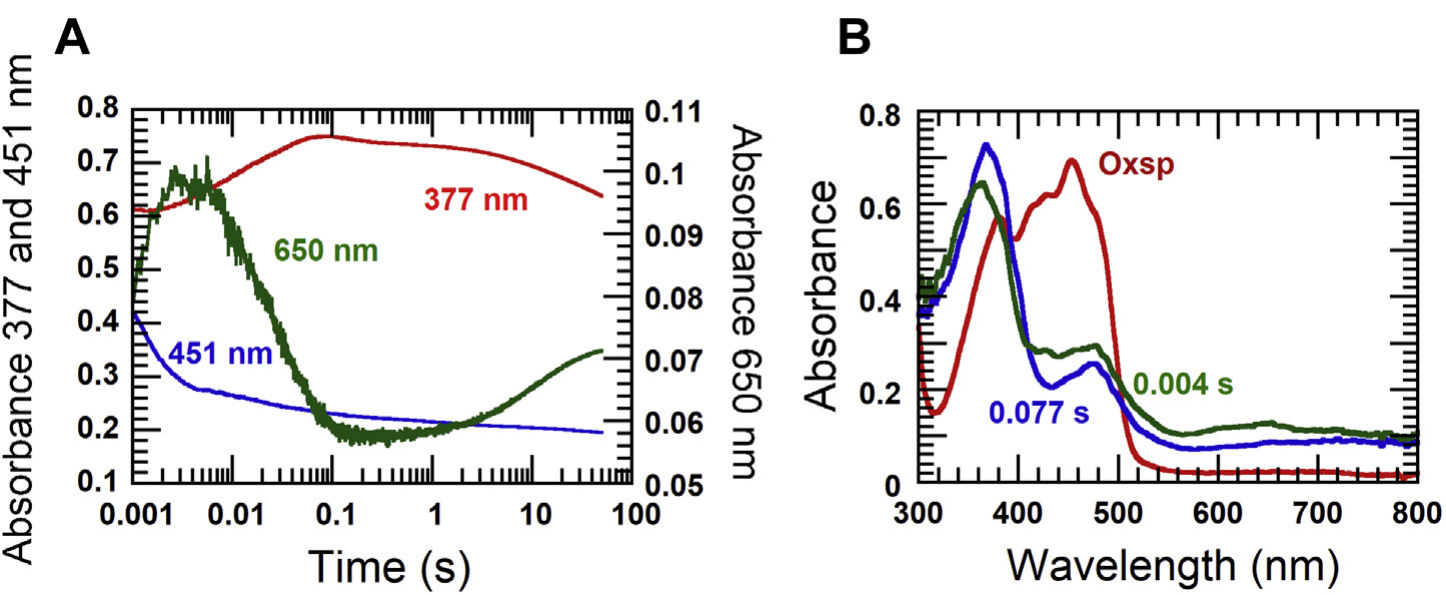
**Reduction of EtfAB with two NADH equivalents.** 34 μM EtfAB was mixed 68 μM NADH. *A*, the reaction was monitored at 451 nm for flavin reduction, 377 nm for the red semiquinone of FAD, and 650 nm for the charge–transfer complex formation. *B*, the oxidized EtfAB (*red line*) was almost completely reduced with NADH within 0.004 s concomitant with a partial formation of the red semiquinone (*green line*). The latter reached its maximum at 0.077 s (*blue line* at 377 nm). EtfAB, Etf (electron transfer flavoprotein) containing α-FAD bound on A- and β-FAD bound on B-subunit.

To investigate the first and second reduction of β-FAD, a solution of 34 μM EtfAB was mixed with two equivalents of NADH (68 μM). Under this condition, only 34 μM β-FAD was reduced, leaving 34 μM NADH in the solution. Kinetic traces of the reaction monitored at 451 nm showed fast reduction, which occurred during the dead time until 0.004 s (*blue line*, Fig. 5*A*) similar to the results observed in Figure 3*A*. A kinetic trace of the reaction monitored at 650 nm (*green line*, Fig. 5*A*) also showed that the charge–transfer complex was formed during this phase. The second phase (0.004–0.077 s) was the decay of the charge–transfer complex (*green line*, Fig. 5*A*) with an increase in the absorbance at 377 nm (*red line*, Fig. 5*A*). The observed rate constant of this phase (47 s^−1^) was similar to that obtained for the decay of charge transfer in the previous section (*red line*, Fig. 3*A*).

This second phase was interpreted as oxidation of β-FADH^−^ by intermolecular and intramolecular electron transfers to two molecules of α-FAD, resulting in two molecules of α-FAD^•−^. The charge–transfer complex exhibited a maximum absorbance of 0.10 at 650 nm (*green lines* in Fig. 5, *A*–*B*). The change from the green to the blue spectrum (Fig. 5*B*) was consistent with the decrease of ΔA_650_ = 0.041, which is equivalent to oxidation of 41% β-FADH^−^ by intramolecular and intermolecular electron transfer to α-FAD.

In the third phase (0.077–50 s), a slow increase of absorbance at 650 nm (*green line*, Fig. 5*A*) occurred concomitantly with a decrease of absorbance at 377 nm and 451 nm with a rate constant of 0.050 ± 0.001 s^−1^ (*red and blue lines*, Fig. 4*A*). This phase was interpreted as the second round of reduction of EtfAB-bound β-FAD by excess NADH to form another charge–transfer complex which is much slower than the formation of the charge–transfer complex during the first phase. These data indicate that the presence of α-FAD^•−^ instead of α-FAD inhibits the rate of β-FAD reduction (Fig. 4*A*). To get a clearer picture of this effect, the reduction kinetics of EtfAB with various NADH concentrations were investigated.

#### Kinetic mechanism of EtfAB reduction by various NADH concentrations and kinetic isotope effects on the reduction kinetics

**Figure 6:**
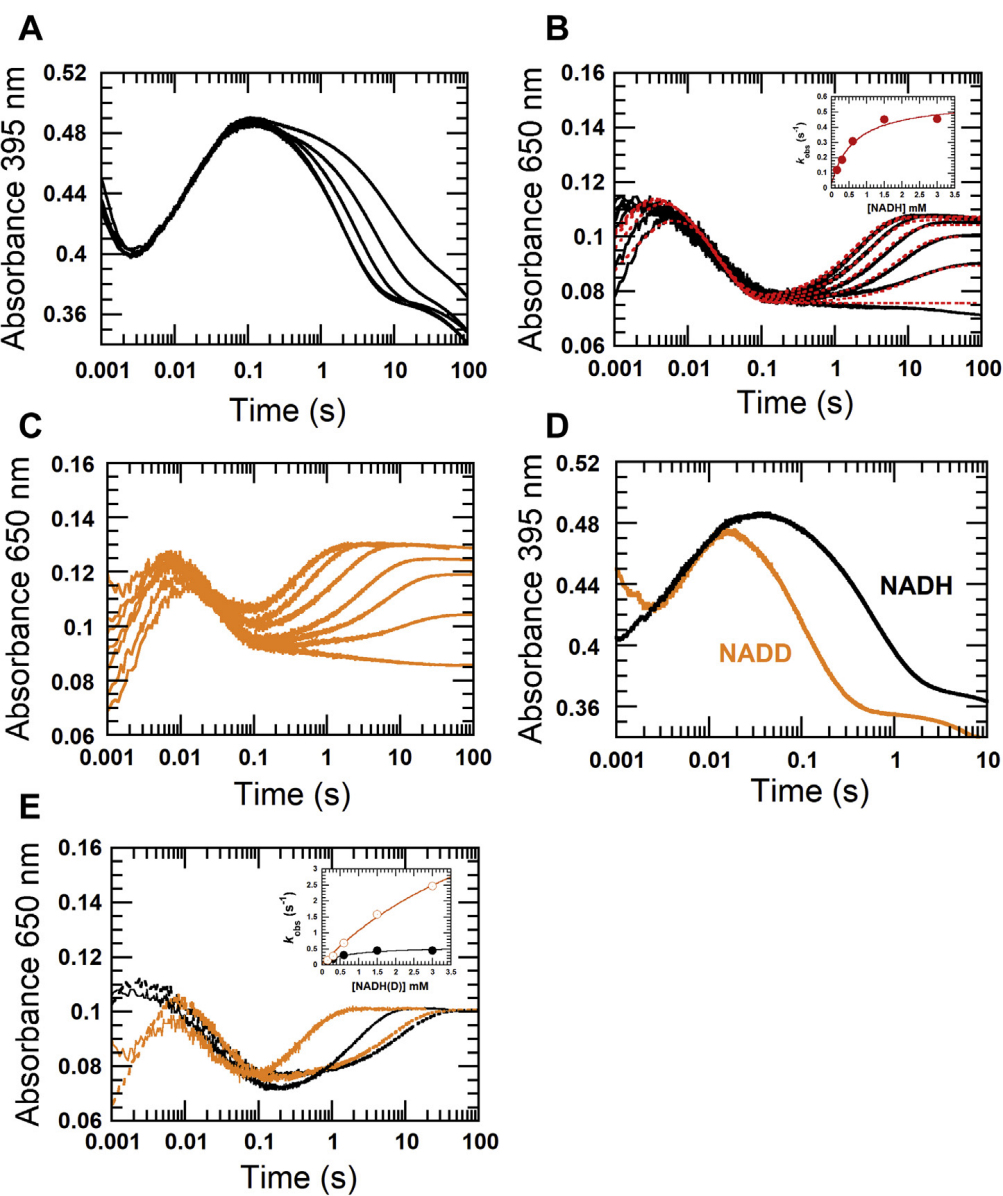
**Reduction of EtfAB with NADH and the kinetic isotope effect using (4*R*)-[4-**^**2**^**H]NADH (NADD).** 36 μM EtfAB was mixed with increasing NADH concentrations, 36 μM, 72 μM, 0.15 mM, 0.3 mM, 0.6 mM, 1.5 mM, 3 mM. *A*, the reactions were monitored at 395 nm for the red semiquinone and *B*, at 650 nm for the charge–transfer complex. The upper to lower kinetic traces were caused by the increasing NADH concentrations as described above (395 nm, *black lines* in *A*), and vice versa at 650 nm (*black lines* in *B*). The fast forming charge–transfer complex represents the first reduction of β-FAD with NADH, whereas the slow forming charge–transfer complex leads to the second reduction. The observed rate constants (*k*_obs_) of the second charge–transfer complex formation exhibit saturation kinetics with increasing NADH concentrations (inset in Fig. 6, *B*). The *red dotted-line* traces are from simulations using the model according to Fig. 7. *C*, the deuterium isotope effect on the reduction of EtfAB was performed using NADD with the same concentrations as with NADH. The reactions were monitored by the absorbance change at 650 nm at the first and second reduction. *D*, the kinetic traces at 395 nm of the red semiquinone formation from the reduction with 3 mM NADD shows the same observed rate constant as with NADH. *E*, the kinetic traces of 3 mM NADH (*black lines*) and 3 mM NADD (*orange lines*) are compared with those of 0.15 mM NADH (*dotted-black line*) and 0.15 mM NADD (*dotted-orange line*). The inset shows the plot of the observed rate constants of the slow reduction forming the second charge–transfer complex. The rate constants using NADD (*open circle-blue line*) are higher than those using NADH (*close circle-red lin*e) at the same concentrations indicating an inverse isotope effect on the observed rate constants of the hydride transfer. EtfAB, Etf (electron transfer flavoprotein) containing α-FAD bound on A- and β-FAD bound on B-subunit; EtfaB, Etf containing only β-FAD

Reduction of EtfAB with various NADH concentrations under pseudo-first order conditions was investigated. To avoid high background absorbance from high NADH concentrations, the absorbance at 395 nm instead of 377 nm was used to monitor formation of the red semiquinone (Fig. 6*A*). The first kinetic phase (dead time −0.003 s) monitored at 395 nm showed a decrease in absorbance because of the reduction of β-FAD which occurred concurrently with an increase in absorbance at 650 nm, indicating that this phase was the formation of the charge–transfer complex (FADH⁻:NAD^+^) (Fig. 6*B*). As most of the first-phase kinetics occurred during the dead time of the instrument, the data obtained for this phase could not be reliably used for analyzing the reaction kinetics. In the second phase (0.003–0.1 s), the red semiquinone (indicated by absorbance at 395 nm) increased with the observed rate constant, *k*_obs_ = 44 ± 1 s^−1^ (Fig. 6*A*). This rate constant was similar to *k*_obs_ = 47 s^−1^ for semiquinone formation by bifurcation *via* intramolecular and intermolecular electron transfer processes using either stoichiometric NADH (Fig. 3*A*) or two-fold NADH (Fig. 5*A*). The observed rate constants of this phase were independent on NADH concentrations (Fig. 6*A*). In the third phase, the excess NADH further reduced EtfAB-bound β-FAD in the presence of α-FAD^•−^ with an increase of the charge–transfer complex at 650 nm (*black line*, Fig. 6*B*).

**Figure 7:**
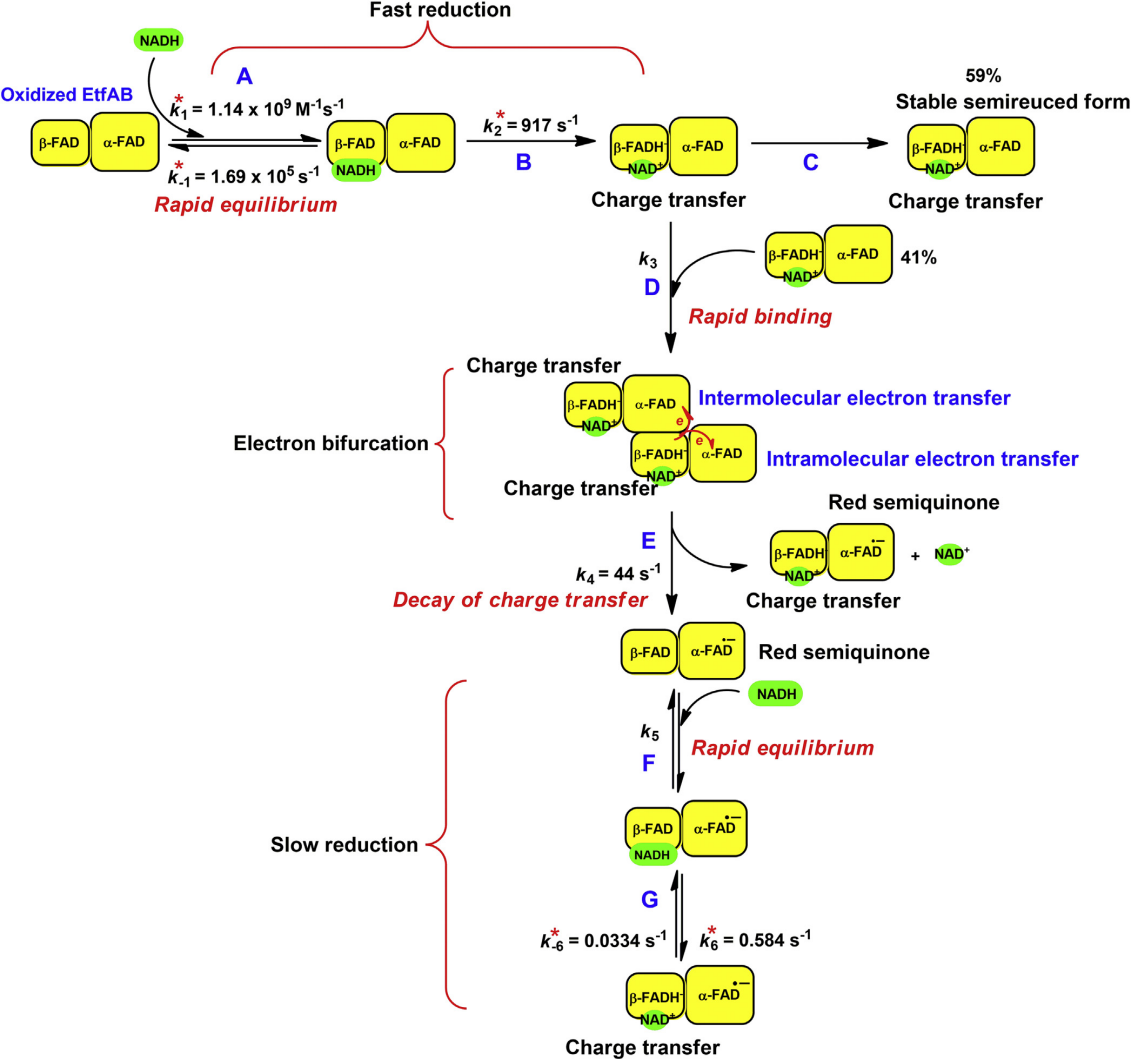
**Kinetic mechanism of the reduction of EtfAB with excess NADH.** The rate constants with asterisks are obtained from kinetic simulations. The reduction of EtfAB with NADH proceeds in a two-step process, rapid equilibrium binding of NADH to oxidized EtfAB forming the Michaelis–Menten complex (*A*), followed by hydride transfer to afford the charge–transfer complex (*B*). Around 59% of total EtfAB remain stable as charge–transfer complex (*C*). The other 41% rapidly bind to another EtfAB to build a complex of two molecules of EtfAB (*D*). One of the two β-FADH^−^ in the complex bifurcates with a rate constant of *k*_4_ = 44 s^−1^, one electron intramolecularly to α-FAD and the other electron intermolecularly to α-FAD of the other EtfAB, leading to two stable molecules of α-FAD^•−^ (*E*). EtfAB with the charge–transfer complex NAD^+^:β-FADH^−^ and α-FAD^•−^ remains stable, whereas EtfAB with β-FAD and α-FAD^•−^ interacts with NADH (*F*) and suffers a second reduction to the charge–transfer complex (*G*). The observed rate constants *k*_3_ (*D*) and *k*_5_ (*F*) were too high to be evaluated. EtfAB, Etf (electron transfer flavoprotein) containing α-FAD bound on A- and β-FAD bound on B-subunit.

Because most of the absorbance change at 650 nm in the first phase mostly occurred during the dead time of the stopped-flow instrument, NADD was used to slow the observed rate constants involved in the first step of the hydride transfer. The kinetic trace of NADD reduction monitored at 650 nm showed that the reaction was indeed slower, and the kinetics of the intermediate charge–transfer formation could be observed during 0.001 to 0.01 s (Fig. 6*C*). The clear observation of a KIE = 2.1 also indicated that the EtfAB-reduction by NADH was specific for the 4*R*-position. The observed rate constants with various NADD concentrations from 30 μM to 3.0 mM showed saturation kinetics, indicating that the first reduction of EtfAB-bound β-FAD by NADH in the presence of α-FAD proceeded with a two-step reduction process, a rapid equilibrium binding of NADH to EtfAB (step A, Fig. 7). The Michaelis–Menten complex was formed with a bimolecular rate constant of *k*_1_ = 1.1 × 10^9^ M^−1^ s^−1^ and a reverse rate constant of *k*_−1_ = 1.7 × 10^5^ s^−1^ followed by hydride transfer to obtain the stable charge–transfer complex of β-FADH^−^:NAD^+^ with a rate constant of *k*_2_ = 917 s^−1^ (step B, Fig. 7). The values of the rate constants of *k*_1_, *k*_−1_, and *k*_2_ were calculated from kinetic simulations (Experimental procedures) developed from the reaction model from step A to B (Fig. 7). Around 59% of the semi-reduced EtfAB remained stable as the charge–transfer complex (step C, Fig. 7), whereas the remaining 41% rapidly transferred electrons intermolecularly and intramolecularly to α-FAD (step D, Fig. 7) with *k*_4_ = 44 s^−1^ to form two different EtfAB species with stable α-FAD^•−^, one with β-FADH^−^ and another with β-FAD (step E, Fig. 7). Two-fold evidence supported that *k*_4_ = 44 s^−1^ was an intrinsic rate constant for intermolecular and intramolecular electron transfer (1). This observed rate constant was not dependent on the NADH concentrations (2). There was no isotope effect on the observed rate constant for semiquinone formation when NADD was used instead of NADH, because an electron rather than a hydride was transferred. The kinetic traces at 395 nm using the highest concentration of 3 mM NADD (*orange line*, Fig. 6*D*) and the same concentration of NADH (*black line*, Fig. 6*D*) showed the same *k*_obs_ = 44 s^−1^ but at a different ΔA_395_.

The EtfAB species containing α-FAD^•−^ and re-oxidized β-FAD (step E, Fig. 7) were again reduced by NADH (second reduction) to reform charge–transfer species (Fig. 5*B*). The kinetics of this reduction were much slower than the first reduction and also showed saturation (inset in Fig. 6*B*), which suggested a two-step process. This included rapid binding of NADH to β-FAD (step F, Fig. 7) followed by hydride transfer to yield the stable charge–transfer complex (step G, Fig. 7). Rate constants associated with the reaction model in Figure 7 were analyzed by kinetic simulations (*red-dotted lines* from simulations *versus solid-black lines* from experimental traces shown in Fig. 6*B*). The hydride transfer rate constant to form the charge–transfer complex of the second reduction was calculated as *k*_6_ = 0.584 s^−1^ and the reverse rate constant as *k*_−6_ = 0.0334 s^−1^ (step G, Fig. 7). The existence of a reversible step was also supported by the fact that each kinetic trace did not reach the same point at the end of the reduction, caused by different equilibrium states according to the NADH concentrations (15). All results of NADD and NADH reductions both indicate that the α-FAD^•−^ state strongly inhibits the reduction rate of β-FAD as compared with the α-FAD state. Actually, the inhibition was *k*_2_/*k*_6_ = 917 s^−1^/0.584 s^−1^ = 1570 fold (steps B and G, Fig. 7).

One interesting observation of the second reduction of β-FAD by NADD, which is different from the first reduction, is the inverse KIE on the charge–transfer complex formation monitored at 650 nm. The first reduction (dead time −0.01 s) exhibited a normal observed KIE ($k_{obs}^{H}/k_{obs}^{D}$) of 2.1 indicated by the first absorption maximum at about 0.006 s with 3 mM NADH (Fig. 6*B*) and 0.01 s with 3 mM NADD (Fig. 6*C*). The second absorption maximum with 3 mM NADH, however, occurred later at around 6 s than that with 3 mM NADD at already 1 s, which indicated an inverse KIE for the second reduction. Furthermore, the kinetic traces in Figure 6*E* after 0.1 s showed that the rates for the second reduction of β-FAD with NADD were greater than those with NADH (orange *versus* black lines). A plot of the observed rate constants from NADD (open-circle orange line) *versus* from NADH (close-circle black line) reactions showed an observed inverse KIE ($k_{obs}^{H}/k_{obs}^{D}$) = 0.19 at the highest NADH and NADD concentrations of 3 mM (inset in Fig. 6*E*), whereas at 30 μM NADH/D, no KIE could be detected.

#### Direct kinetic isotope effects on the second flavin reduction

As the second reduction of β-FAD using NADD showed an inverse KIE on the reduction rate constant which is quite unusual for hydride transfer from NAD(P)H/D to flavin, we further investigated this issue by directly measuring the KIE of the second reduction. For the experiments in Figures 5 and 6, NADH/D was added into the enzyme solution when both FADs of EtfAB were in the oxidized state. The KIE of the second flavin reduction observed in Figure 6 could be because of the combined effects from the first reduction. To directly measure an inverse isotope effect of the second flavin reduction, we carried out the experiment using a double-mixing stopped-flow spectrophotometer. In the first mixing, NADH was added stoichiometrically to reduce β-FAD and generate α-FAD^•−^. Then, NADD was added, and the second reduction was directly observed.

**Figure 8:**
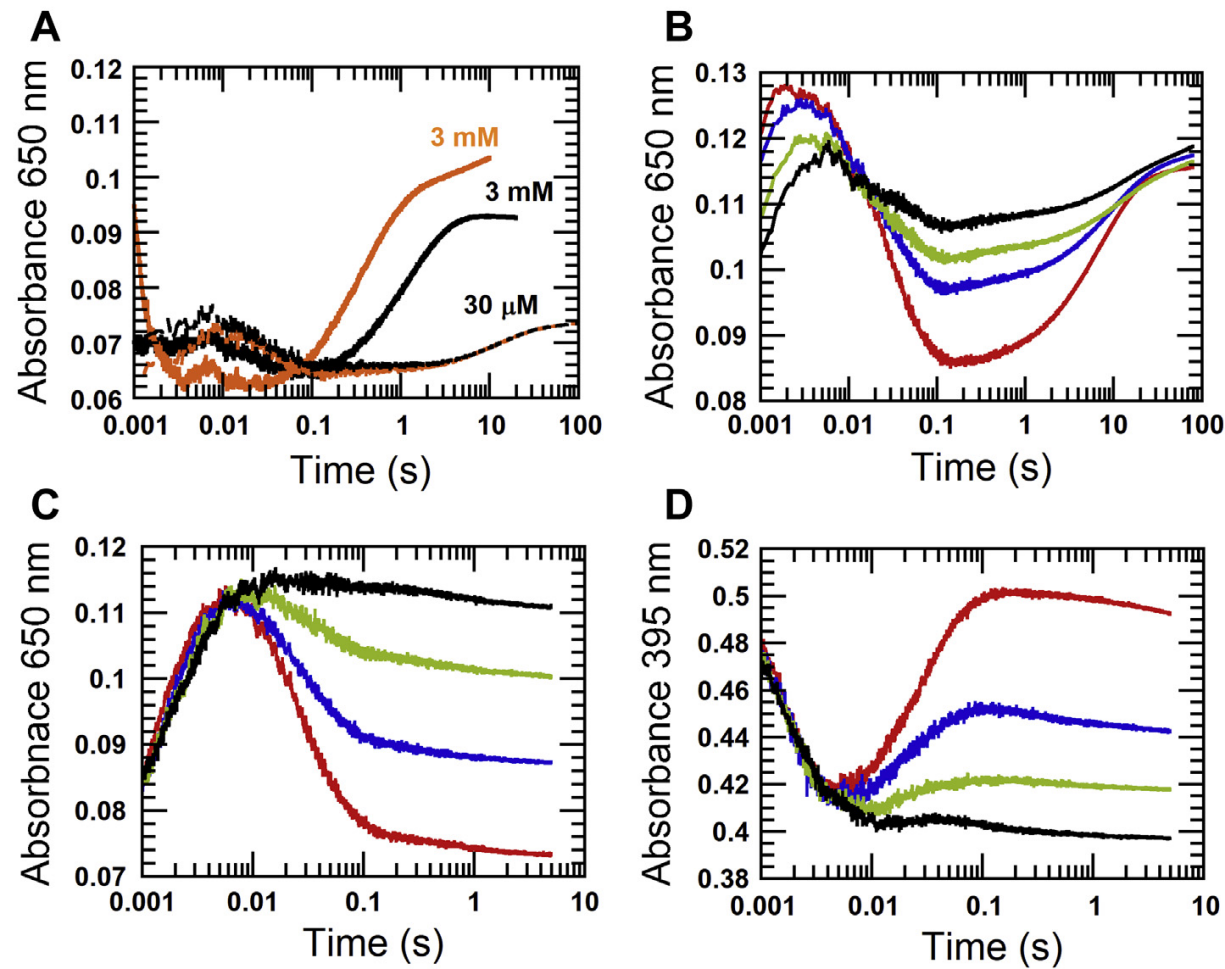
**Influence of NADH(D) and NAD**^**+**^**on the inverse isotope effect.***A*, the kinetic traces are from double-mixing experiments. 60 μM EtfAB was reduced with 60 μM NADH in the first mixing with an age time of 0.1 s to allow for the *red* semiquinone formation (α-FAD^•−^). Then β-FAD of EtfAB containing α-FAD^•−^ was reduced with 30 μM NADH (*black line*) or 30 μM NADD (*orange line*) in the second mixing. Both kinetic traces are compared with those of 3 mM NADH (*black line*) or 3 mM NADD (*orange line*), indicating an inverse isotope effect on the observed rate constants of the hydride transfer to β-FAD, which only occurs in the presence of α-FAD^•−^. *B*, the kinetic traces from mixing of 36 μM EtfAB with 150 μM NADH plus different concentrations of NAD^+^ of 0 μM (*red line*), 150 μM (*blue line*), 500 μM (*green line*), and 1 mM (*red line*). The reactions were monitored at 650 nm to detect a charge–transfer species after the first and the second reduction of β-FAD. *C*, the reduction of 36 μM EtfAB with 36 μM NADH equivalent to β-FAD (NADH) plus the same various NAD^+^ concentrations: 0 μM (*red line*), 150 μM (*blue line*), 500 μM (*green line*), and 1 mM (*black line*). The reactions were monitored at 650 nm for charge–transfer formation and decay, whereas (*D*) the red semiquinone formation was monitored at 395 nm. EtfAB, Etf (electron transfer flavoprotein) containing α-FAD bound on A- and β-FAD bound on B-subunit; NADD, (4*R*)[4-^2^H]NADH.

The experiment was performed by reducing 60 μM EtfAB with 60 μM NADH in the first mixing. The reaction was incubated for 0.1 s to form the stable α-FAD^•−^ and then mixed with 30 μM of either NADH (*black line* indicated as 30 μM, Fig. 8*A*) or NADD (*orange line* indicated as 30 μM, Fig. 8*A*) in the second mixing. The second reduction was monitored at 650 nm to follow the formation of the charge–transfer complex. Both kinetic traces did not show a clear inverse KIE at the low concentration of 30 μM NADH(D) because both reactions gave similar observed rate constants of 0.070 ± 0.001 s^−1^ for NADH and 0.069 ± 0.006 s^−1^ for NADD. When the concentrations of NADH (*black line* indicated as 3 mM, Fig. 8*A*) and NADD (*orange line* indicated as 3 mM, Fig. 8*A*) were raised to 3 mM in the second mixing, the inverse isotope effect was detected similar to the results from the single mixing. The observed rate constants at 3 mM NADD from single mixing *versus* double mixing mode are 2.47 *versus* 2.26 s^−1^, respectively. Therefore, the inverse KIE for the reduction of β-FAD in EtfAB with α-FAD^•−^ bound is an intrinsic property of the second reduction.

#### Identification of the effects of excess NAD^+^ on the reduction kinetics

Another interesting kinetic property of EtfAB is the slowdown of second reduction of β-FAD when α-FAD is at the semiquinone state (α-FAD^•−^) (Fig. 6*B*). To rule out whether this effect might be because of the presence NAD^+^ of (either from dissociation of the β-FADH^−^:NAD^+^ complex or from contamination in the solution of NADH), excess NAD^+^ was added to test its effects on the reduction kinetics of EtfAB. The reduction of 36 μM EtfAB with 150 μM NADH was performed in the single-mixing stopped-flow spectrophotometer in the absence and presence of 150 μM (*blue line*), 500 μM (*green line*), and 1 mM (*red line*) NAD^+^ (Fig. 8*B*) while monitoring the formation of the charge–transfer species at 650 nm (*red line*, Fig. 8*C*). The results clearly showed that the presence of NAD^+^ only slowed down the rate of NADH reduction on the first phase (possibly by competing with NADH), the observed rate constant ∼630 s^−1^ (*red line*, Fig. 8*B*) *versus* ∼240 s^−1^ (*black line*, Fig. 8*B*), and stabilized the charge–transfer species formed. The higher NAD^+^ concentrations prevented charge–transfer decay (Fig. 8*C*), thus decreasing a single electron transfer process to form a semiquinone observed at 395 nm (Fig. 8*D*) on the second step. This indicates that the presence of extra NAD^+^ could only affect the first and second kinetic phases.

For the third kinetic phase (0.1–20 s, Fig. 8*B*) representing the second reduction of β-FAD after electron bifurcation, kinetics of this phase (rate constant of 0.144 s^−1^) was not affected by excess NAD^+^ concentrations. Only the magnitude of the absorbance at 650 nm due to the stabilization of the charge–transfer species was increased (Fig. 8*B*). Therefore, it can be concluded that the slowdown of the second reduction of when α-FAD is in the semiquinone state is indeed an intrinsic property of EtfAB. The data also clearly indicate that the different redox status of α-FAD bound on EtfAB can control the reduction kinetics of β-FAD bound on the other subunit.

#### Reduction of β-FAD with NADH in the presence of α-FADH^−^ and α-FAD^•^⁻prepared with dithionite

**Figure 9:**
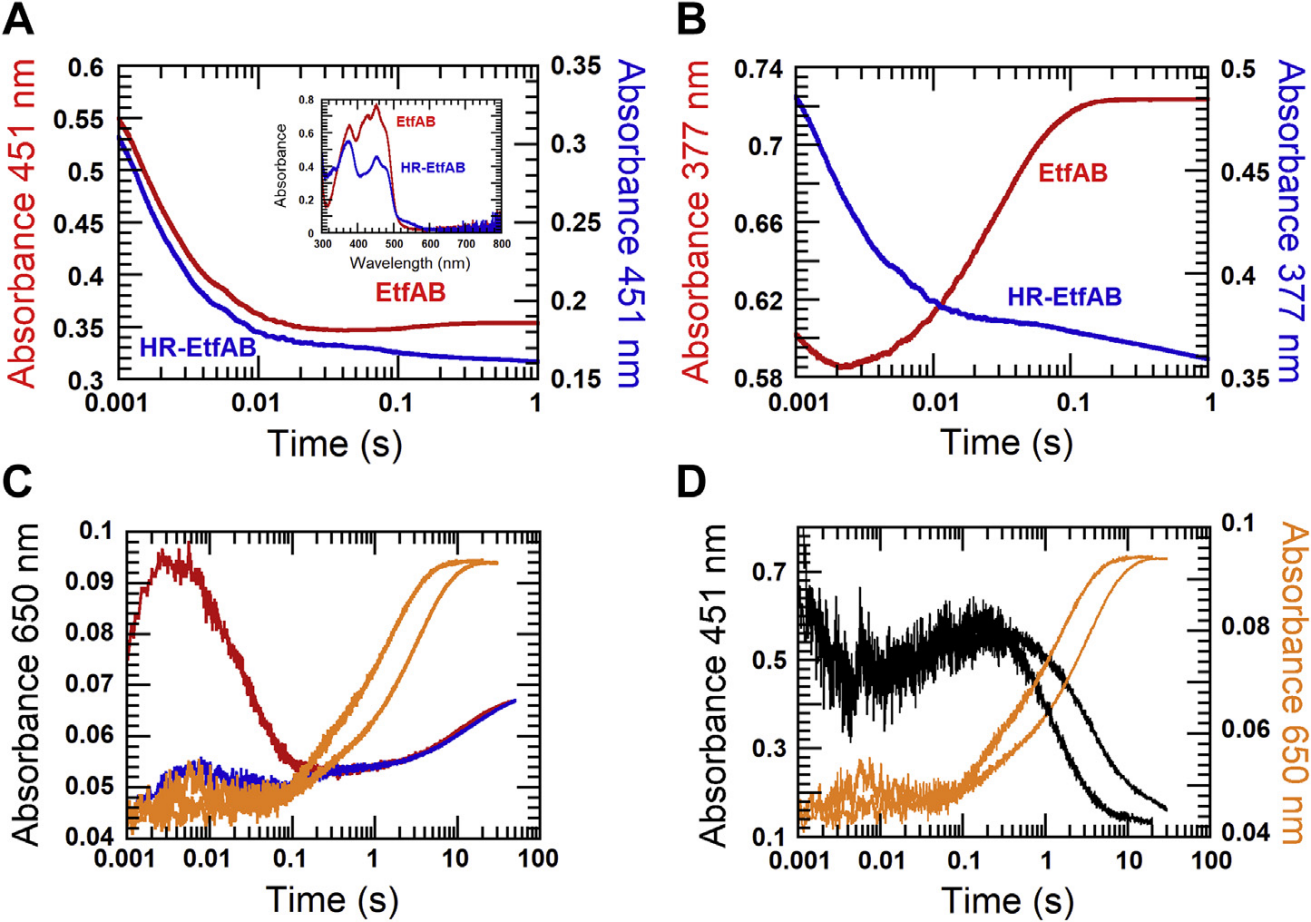
**The reduction of β-FAD in the presence of α-FADH**^**−**^**and in the presence of α-FAD**^**•−**^**.***A*, α-FADH⁻ in 36 μM EtfAB (HR-EtfAB) was prepared by titration of the oxidized enzyme (*red line*, *inset*) with sodium dithionite under anaerobic conditions until the absorption at 377 nm remained constant (*blue line, inset*). The HR-EtfAB solution was mixed with 36 μM NADH in a stopped-flow spectrophotometer and monitored at 451 nm for flavin reduction (*blue line*). The kinetic trace showed high similarity to that from the reduction of 36 μM oxidized EtfAB with 36 μM NADH (*red line*). *B*, the reductions of oxidized EtfAB (*red line*) and HR-EtfAB (*blue line*) were monitored at 377 nm. *C*, α-FAD in 36 μM EtfAB was reduced to α-FAD^•−^ by titration with sodium dithionite to form the red semiquinone with a peak at 377 nm. The reductive titration was paused until the peak at 377 nm reached a stable point. The solution was mixed with 36 μM NADH in a stopped-flow spectrophotometer, and the reduction of β-FAD was monitored at 650 nm for the charge–transfer complex (*blue line*). This kinetic trace is superimposed with the second reduction of β-FAD in 36 μM EtfAB containing α-FAD with 68 μM NADH at the same wavelength (*red line*). In two further experiments, 36 μM EtfAB containing α-FAD^•−^was mixed with 0.75 mM NADH (*right orange line*) or 3 mM NADH (*left orange line*). *D*, o*range lines* as in C. The *black lines* show that β-FAD is reduced to the hydroquinone with NADH (right 0.75 mM and left 3 mM). EtfAB, Etf (electron transfer flavoprotein) containing α-FAD bound on A- and β-FAD bound on B-subunit.

The results from Figure 5*A* and Figure 6*B* have demonstrated that the semiquinone state (α-FAD^•−^) inhibits the second reduction of β-FAD 1570-fold. To show whether this effect might be because of the negative charge or to the radical, 36 μM half-reduced EtfAB (HR-EtfAB) containing 36 μM β-FAD and 36 μM α-FAD-hydroquinone (α-FADH^−^) was applied. The previous report of the reductive titration of EtfAB showed that both reduction potentials of semiquinone formation and reduction of α-FAD are much higher than the two-electron reduction potential of β-FAD (11). Therefore, HR-EtfAB could be prepared by reductive titration with sodium dithionite (inset in Fig. 9*A*) in a tonometer under nitrogen gas at positive pressure and monitored using a diode array spectrophotometer. The solution of HR-EtfAB was mixed with 36 μM NADH. The reduction of HR-EtfAB containing β-FAD and α-FADH^−^ was monitored at 451 nm for flavin reduction (*blue line*, Fig. 9*A*). The observed rate constant of the reductive phase (dead time −0.02 s) was ∼390 s^−1^, which was similar to the observed rate constant for the reduction of β-FAD when α-FAD was in the oxidized form (*red line*, Fig. 9*A*). The results clearly show that the radical rather than the negative charge of the radical anion of α-FAD was responsible for the inhibition. The kinetic traces of the HR-EtfAB preparation did not show a significant increase in the absorbance at 377 nm because of remaining oxidized α-FAD (*blue line*, Fig. 9*B*), as compared with the reduction of oxidized EtfAB (*red line*, Fig. 9*B*).

Another experiment showed that the inhibition of the reduction of β-FAD by α-FAD^•−^ was independent of the preparation of the semiquinone, either by the second reduction of EtfAB with NADH or by direct reduction of α-FAD to α-FAD^•−^ with dithionite. EtfAB (36 μM) was titrated with dithionite until the absorbance at 377 nm remained stable (11). The solution with the formed semiquinone was mixed with 36 μM NADH. The reduction of β-FAD with NADH in the presence of α-FAD^•−^ was monitored at 650 nm for the charge–transfer complex (*blue line*, Fig. 9*C*). The kinetic trace shows a slow reduction with an increase in absorbance at 650 nm (0.1–50 s), which is the same as the slow second reaction of β-FAD in 36 μM EtfAB, when 72 μM NADH (two-fold of β-FAD) was used (*red line*, Fig. 9*C*). The right and left *orange lines* in Figure 9*C* represent experiments, in which 0.75 mM and 3 mM NADH were applied, respectively, compared with Figure 6*B*. The kinetic trace with 3 mM NADH ended at 10 s which is similar to the second reduction of EtfAB by the same NADH concentration (uppermost trace, Fig. 6*B*). The kinetic traces at 451 nm for β-FAD reduction (*black lines*, Fig. 9*D*) show the inverse kinetic properties as those at 650 nm. These results confirm that the intrinsic properties of the radical of α-FAD, not the negative charge, control the slow reduction of β-FAD, even when NADH was not the reducing agent for semiquinone formation.

## Discussion

This report describes the first extensive investigation of a bifurcating EtfAB using rapid kinetics. Although the reduction kinetics of the closely related EtfAB from *Megasphaera elsdenii* with NADH were investigated earlier (16), the data reported here could not be obtained, because the dead time of the used stopped-flow instrument was only 0.1 s. With the limitation of such a long dead time, a great deal of the information on the reduction kinetics before the red semiquinone state such as those shown in Figures 1*C* and 6*B* were missing.

**Figure 10:**
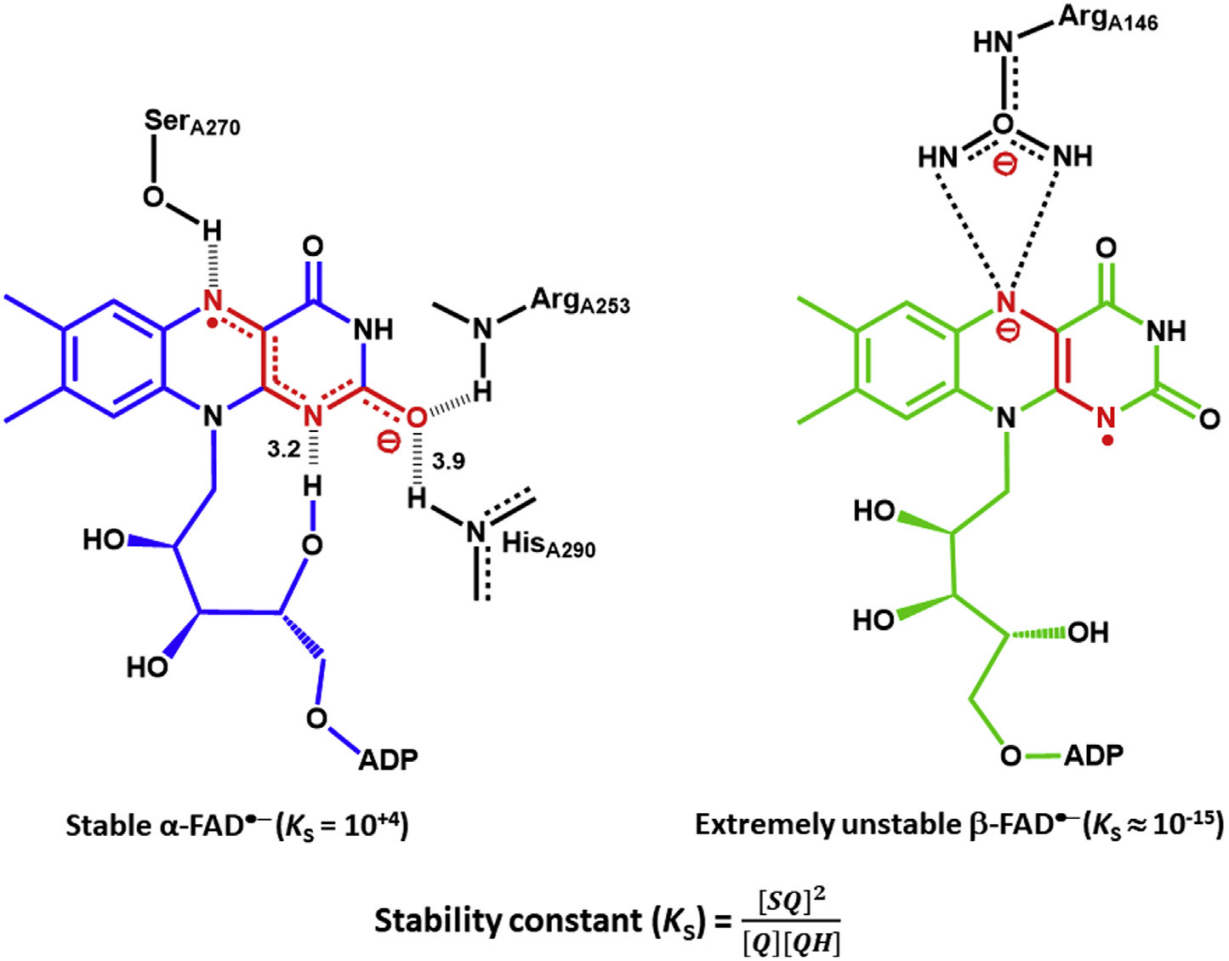
**Interactions of the stable α-FAD**^**•**^**⁻ and the extremely unstable β-FAD**^**•**^**⁻ with the surrounding amino acid side-chains.** Like in flavodoxin, in α-FAD^•−^ the 4'-hydoxyl group of the ribityl moiety is hydrogen bonded to N1 of the isoalloxazine ring. Between O2 and N5, the radical can be resonance stabilized over six atoms. Both effects contribute to the semiquinone stability. In β-FAD^•^⁻, there is no resonance stabilization of the radical, because the positive charge of Arg 146 fixes the negative charge at N5 (7). The low stability constant of β-FAD^•^⁻ is required for electron bifurcation (2, 5).

An intriguing property of the EtfAB system reported here is the significant effect of the occupancy and oxidation state of α-FAD on the functional and electronic properties of β-FAD. The crystal structure of EtfAB shows that α-FAD is located on the rotatable domain II, 18 Å apart from β-FAD. In the presence of Bcd, this distance is extended up to 30 Å, whereas in its absence, α- and β-FAD can approach each other up to 14 Å (7, 10). EtfAB without α-FAD, called EtfaB, indicated that α-FAD stabilized the conformation of the β-subunit in EtfAB to become a more homogeneous population (Fig. 1*C* *versus* Fig. 3*A*). In the absence of α-FAD, an equilibrium existed between a fast (EtfaB∗) and a slow reacting population (EtfaB), which reacted separately with NADH. In the presence of α-FAD, the fast reacting species became the major species. The peak of the charge–transfer complex, β-FADH^−^:NAD^+^, was shifted from 750 nm in EtfaB, (Fig. 1*A*) to 650 nm in EtfAB (Fig. 3*B*). That the regions around N(5), C(4a), C(10a), and N(1) atoms of the isoalloxazine ring mainly contributed to the formation of the charge–transfer complex was demonstrated by studies of monomeric sarcosine oxidase with substrate analogs using Raman spectroscopy (17). For the blue shift of the charge–transfer absorption band observed with EtfAB, the α-FAD-induced changes of the interactions of these atoms with amino acid side-chains (see Fig. 10) could be responsible. A study of the interaction between acyl-CoA dehydrogenase and substrate analogs showed a correlation between the distance of flavin to ligand and the shift of the charge–transfer band. The longer distance gives the longer wavelength maximum of the charge–transfer band (18, 19). Therefore, the hypsochromic shift of the charge–transfer band in EtfAB, possibly stems from a shorter distance between β-FADH^−^ and NAD^+^ in the presence of α-FAD. Interestingly, only the ADP part of NAD^+^ could be localized in the crystal structure of EtfAB, indicating that binding of NADH near β-FAD must induce a conformational change (7). Consequently, the distances between β-FAD and NADH as well as between β-FADH^−^ and NAD^+^ are variable and dependent on the overall conformation of EtfAB.

Owing to the absence of Fd, a part of the charge–transfer complex of β-FADH^−^ with NAD^+^ remained stable and another part acted as alternative electron acceptor (Fig. 7). Electrons from β-FADH^−^ bifurcated intramolecularly to α-FAD of the same EtfAB molecule and intermolecularly to α-FAD of another EtfAB molecule, whereby the stable anionic red semiquinone of α-FAD (α-FAD^•−^) at 377 nm got its maximum value (Fig. 4). The intermolecular electron transfer agrees well with previous suggestions based on the enzyme structures (7, 20). Addition of EtfaB was expected to inhibit the formation of α-FAD^•−^ because of the dilution of the acceptor, but the opposite happened (Fig. 3*C*). Apparently, the concentration of the donor β-FADH⁻ was the rate limiting factor, which increased by adding EtfaB, whereby the concentration of α-FAD remained constant. Thus β-FADH⁻ on EtfaB bifurcated intermolecularly to two EtfAB molecules, which raised the question whether these electron transfers could be regarded as energetic bifurcation, because the acceptors α-FAD were identical and comprised the same reduction potential.

One interesting finding in this report was the control of the reactivity of β-FAD by the presence and redox state of α-FAD. The comparison of the reduction kinetics of EtfaB and EtfAB with stoichiometric NADH indicated that the presence of α-FAD increased the rate of β-FAD reduction about two-fold (Fig. 3*A* *versus* Fig. 1*B*). The charge–transfer complex (β-FADH^−^:NAD^+^) of EtfaB reached its stable value at 750 nm (ε = 3.1 mM^‒1^ cm^‒1^) around 0.010 s, whereas the charge–transfer complex of EtfAB passed its highest peak at 650 nm (ε = 3.04 mM^‒1^ cm^‒1^) already at 0.006 s. The observed rate constant of the reduction of β-FAD with NADH in the presence of α-FAD was around 920 s⁻^1^, whereas in the presence of α-FAD^•^⁻, the rate constant decreased to 0.58 s^−1^ by a factor of 1600 (*k*_2_/*k*_6_, Fig. 7). Apparently, the vicinity of the α-FAD^•−^ anion radical at a distance of 14 to 18 Å (7) inhibits the hydride transfer from NADH to β-FAD. The cause of the inhibition could either be the radical or the negative charge. EtfAB, in which α-FAD was converted with dithionite to the hydroquinone (FADH⁻), revealed exactly the same rate of reduction with NADH as that with α-FAD in the oxidized form (Fig. 9*A*). Hence, the cause of the inhibition must be the radical but not the negative charge of the semiquinone. The experiment also shows that small conformational changes induced by the conversion of FAD to FADH⁻ have no impact on the reduction rate. Further experiments demonstrate that it is the radical and not the mode of its formation, either by NADH or dithionite, which acts as inhibitor (Fig. 9, *C*–*D*). The question arises of how can a radical inhibit a hydride transfer at a distance of 14 to 18 Å? The authors are not aware of any enzyme, in which a substrate or a cofactor is reduced by NAD(P)H in close vicinity of a radical. A reason could be a radical-induced large decline of the reduction potential of β-FAD to a value much lower than that of NAD^+^ (−320 mV). Because EtfAB with β-FAD and α-FAD^•−^ is reduced with NADH (Fig. 6), albeit slowly, the reduction potential cannot differ significantly from that of a EtfAB with β-FAD and α-FAD (−270 mV) (11).

The most interesting observation of the hydride transfer reaction is the inverse KIE. The deuterium KIE of these reactions at 3 mM NADH/NADD changed from $k_{obs}^{H}/k_{obs}^{D}$ = 2.1 of the first reduction (fast process) to ≥0.19 of the second reduction (slow process). This unexpected very large inverse deuterium KIE, which decreased from 1.0 at 30 μM NADD to 0.19 at 3 mM NADD cannot be readily explained. A similar model for an inverse isotope effect on hydride transfer, but not in a biological system, are the reductions of carbocations by metal hydrides. Here, the same picture arose, the lower the rate the lower the KIE; values between 2 and 0.47 have been observed (21).

The hydride transfer from NADH to FAD possibly proceeds over a very short distance between C4 of NADH and N5 of FAD, most likely within a van der Waals contact, which can be regarded as quantum tunneling. The spin of the radical could directly interact with the tunneling hydride. Because a deuteride transfer is less associated with tunneling (22), its inhibition by radical interactions will not be as pronounced as that of a hydride transfer and could result in a higher rate with NADD than with NADH. The authors are aware that this large inhibition of the hydride transfer from NADH to with a high inverse deuterium isotope effect by the nearby presence of α-FAD^•−^ has no precedence, and the explanation of this phenomenon is very speculative.

The inhibition of the reduction of β-FAD with NADH by the adjacent α-FAD^•−^ has implications on the mechanism of electron bifurcation. The question, whether α-FAD^•−^ or α-FADH^−^ transfers one electron further to Bcd, can be resolved. From a kinetic point of view, one would favor α-FAD^•−^, because NADH reduces β-FAD in the presence of α-FAD at a high rate (Fig. 7). After electron bifurcation, however, the electron transfer from FAD^•−^ to Bcd is thermodynamically difficult, because the stable α-FAD^•−^ has the high reduction potential of $E^{{}^{\circ}}\prime$ = +135 mV (11). Alternatively, NADH reduces β-FAD in the presence of α-FAD^•−^, yielding α-FADH^−^ after bifurcation. Owing to the lower reduction potentials, $E^{{}^{\circ}}\prime$ = −36 mV/−228 mV (11), α-FADH^−^ transfers one electron much easier to δ-FAD of Bcd, and the reformed α-FAD^•−^ gets again an electron from the next round of bifurcation. Hence, all reductions of β-FAD with NADH occur in the presence of α-FAD^•−^ (Fig. 1 in ref. (11)). If this reduction is the rate-limiting step, electron bifurcation should proceed at *k*_6_ = 0.6 s^−1^ (4 °C, Fig. 7). Incubation of EtfAB, Bcd, Fd, and NADH in a cuvette at 25 °C gave a specific activity of 3 μmol NADH × mg^−1^ EtfAB × min^−1^ (7) from which a turnover number of about 3 s^−1^ can be derived (EtfAB, m = 66 kDa). Thus, the rates are comparable and lead to the conclusion that the reduction of β-FAD with NADH in the presence of α-FAD^•−^ indeed represents the rate-limiting step of electron bifurcation. It is still not clear why nature chose to conduct the Etf reduction with such a slow rate. Probably, the physical properties of this system have not allowed to make it faster. Nevertheless, the rate is still fast enough for electron bifurcation to occur and to serve the physiological needs.

This interaction between the two flavins is certainly not restricted to the well-characterized EtfAB-Bcd systems from *A. fermentans* (7), *M. elsdenii* (8), *Clostridium kluyveri* (23), and *Clostridium difficile* (10) because there are other systems in which homologues of EtfAB bifurcate electrons to low potential Fd or Fld and to the high potential acceptors, as are caﬀeyl-CoA (caﬀeyl-CoA reductase, CarC from *Acetobacterium woodii* (24)), pyruvate (lactate dehydrogenase, LctD from *A. woodii* (25)), and ubiquinone (ubiquinone reductases, Etf/FixC from *Azotobacter vinelandii* (26), *Rhodopseudomonas palustris* (27), and *Pyrobaculum aerophilum* (18)). All these systems contain β-FAD and α-FAD in the highly conserved arrangement as in *A. fermentans* Etf (6). Thus, the reduction of β-FAD with NADH in the presence of α-FAD^•−^ is common to all the bifurcating Etfs of anaerobic bacteria, anaerobic compartments of aerobes, and anaerobic archaea.

In conclusion, we report for the first time the kinetic mechanisms of a bifurcating Etf which reveals several intriguing features regarding the control of FAD reduction by a neighboring FAD. The findings here will serve as a basis for future studies to explain how the reactivity of this new class of flavoproteins can be controlled and linked with other electron acceptors. Whether a radical can interfere with hydrogen tunneling emerged as an exciting question and requires further investigation.

## Experimental procedures

### Reagents

NADH (purity ≥ 95%), FAD (purity ≥ 95%), FMN (purity ≥ 93%), NAD^+^, and imidazole were purchased from Tokyo Chemical Industry (Tokyo, Japan). Guanidinium hydrochloride (GuHCl) was purchased from Merck (Calbiochem) (Darmstadt, Germany). Chromatographic medias (DEAE-Sepharose, G-25 Sephadex, IMAC-Sepharose) were purchased from GE Healthcare (Uppsala, Sweden). AG MP-1M (macroporous anion exchange resin) was purchased from Bio-Rad (Hercules, CA). Deuterated formic acid (98%) was purchased from Cambridge Isotope Laboratories (Tewksbury, MA). Concentrations of the following compounds were determined using the known absorption coefficients: NADH, ε_340_ = 6.3 × 10^3^ M^−1^ cm^−1^ (28); FAD, ε_450_ = 11.3 × 10^3^ M^−1^ cm^−1^ at pH 7.0. All component parts and enzyme solutions were placed in an anaerobic glove box for 30 min or exchanged gas using an anaerobic train to equilibrate the solutions with nitrogen gas before use.

### Preparation of EtfAB and EtfaB

The gene of EtfAB in the vector pET-11a was induced for protein production using the autoinduction medium (29). Growth of microorganisms and protein purification were carried out according to the previously described protocols (11). The EtfaB was prepared from EtfAB by removing FAD from the A-subunit using a phenyl Sepharose column and KBr as previously reported (11). The purified EtfAB and EtfaB were exchanged into 50 mM potassium phosphate pH 7.0. The concentrations of EtfAB (two FAD per α- and β-subunits (one for each subunit)) and EtfaB (one FAD per α- and β-subunits) were determined using the molar absorption coefficients of ε_451_ = 10.5 × 10^3^ M^−1^ cm^−1^ and ε_452_ = 11.2 × 10^3^ M^−1^ cm^−1^ (11) before storing the enzyme solutions at −80 °C freezer. The EtfAB was thawed, and 200 μM FAD was added to ensure that all subunits were saturated with FAD. The excess FAD was removed by passing through a Sephadex G-25 column to exchange the enzyme solution into 50 mM potassium phosphate pH 7.0 before using in experiments.

### [4R-^2^H]NADH synthesis

NADD was synthesized by mixing 50 mM [^2^H]formic aid (adjusted to pH 8.5), 0.1 g NAD^+^, and 40 units of formate dehydrogenase in 100 mM sodium bicarbonate pH 8.5 in a total volume of 12 ml. The mixture was incubated at room temperature with stirring for 3 h in the dark. The reaction was monitored for completeness by measuring absorbance at 340 nm. The reaction mixture was loaded onto an anion exchange resin (AG MP-1M, chloride form) column (*ϕ* 1.5 cm × 12 cm) equilibrated with 400 ml freshly prepared 0.5 M ammonium carbonate pH 9.0. The column was washed with 500 ml of 0.5 M ammonium carbonate pH 9.0, and NADD was eluted with 300 ml of 1 M ammonium carbonate pH 9.0. The purity of NADD was determined based on the ratio of A_260_/A_340_ ∼ 2.26. To the pooled fractions, solid ammonium sulfate was added to 10% saturation. This solution was loaded onto a C18 Sep-Pak (C18 6 ccVac Cartridge 5 g) from Waters (Milford, MA) equilibrated with 1 M ammonium carbonate and 10% ammonium sulfate pH 9.0 (25 ml). The Sep-Pak column was washed with 25 ml of 120 mM Tris-HCl pH 8.0 to remove extra salts. The NADD fractions were eluted with 25 ml of absolute ethanol. Ethanol was removed using a rotary evaporator until the volume of solution was ∼5 to 6 ml. The concentration of NADD was determined by its absorption spectrum. The yield was 6 ml of ∼25 mM NADD. The solution was kept at −30 °C until use.

### Stopped-flow measurements under anaerobic conditions

Anaerobic experiments were performed using a TgK Scientific (Bradford-on-Avon, UK) Model SF-61DX stopped-flow spectrophotometer in both single-mixing and double-mixing mode. The stopped-flow apparatus was made anaerobic by flushing the flow system with an anaerobic buffer solution containing 0.5 mg/ml dithionite in 100 mM potassium phosphate pH 7.0 and equilibrating the system in the same anaerobic buffer overnight. The anaerobic buffered dithionite solution was prepared in 100 mM potassium phosphate pH 7.0 equilibrated with nitrogen (high purity > 99.9%) in a closed tonometer by alternating between evacuation and equilibration for forty cycles using a three-way oblique manifold connected to an anaerobic train. The anaerobic buffer was kept in the tonometer under positive pressure nitrogen gas and was mixed with solid dithionite in the side arm. Before the experiments were performed, the dithionite in the flow system of the stopped-flow instrument was removed by washing the flow system three times with anaerobic 50 mM potassium phosphate pH 7.0

All stopped flow measurements were performed in anaerobic 50 mM potassium phosphate pH 7.0 at 4 °C. The described concentrations were those obtained after mixing. The dead time of the stopped flow instrument was 1 to 2 ms. The reactions were monitored using a charge coupled device array detector.

### Kinetic analysis and simulations

Observed rate constants (*k*_obs_) were obtained from the kinetic traces using exponential fits and the software packages Kinetic Studio (Hi-Tech Scientific, Salisbury, UK) and Program A (written at the University of Michigan by Rong Chang, Jung-yen Chiu, Joel Dinverno, and David P. Ballou).

Observed rate constants from reactions of enzymes with substrates under pseudo-first order conditions ([substrate] >> [enzyme]) were plotted *versus* substrate concentrations to analyze for the kinetics mechanism using Marquardt–Levenberg nonlinear fit algorithms included in KaleidaGraph (Synergy Software 4.5). For electron transfer reactions between proteins, the pseudo-first order condition could not be applied because of high absorption background and concentration limitation. The reaction mechanism models and rate constants were obtained from kinetic simulations using the enzyme kinetic software KinTek Explorer 9 (30, 31).

## Data availability

All the data are in the manuscript.

## Conflict of interest

The authors declare that they have no conflicts of interest with the contents of this article.
